# CTLA4-Ig Abatacept Ameliorates Proteinuria by Regulating Circulating Treg/IL-17 in Adriamycin-Induced Nephropathy Rats

**DOI:** 10.1155/2020/2347827

**Published:** 2020-04-25

**Authors:** Lanjun Shuai, Qia Cheng, Tian Shen, Zhuwen Yi, Xiaochuan Wu

**Affiliations:** ^1^Department of Pediatrics, The Second Xiangya Hospital of Central South University, Changsha, Hunan 410011, China; ^2^Laboratory of Pediatric Nephrology, Institute of Pediatrics, Central South University, Changsha, Hunan 410011, China

## Abstract

**Objective:**

This study is aimed at investigating the efficacy of CTLA4-Ig abatacept in normalizing proteinuria and its possible mechanism in adriamycin-induced nephropathy (AIN) rats.

**Methods:**

A total of 32 healthy male Sprague-Dawley rats were randomly divided into a normal group, an AIN group, an abatacept group, and a prednisone group. Adriamycin (6.5 mg/kg) was injected once via the tail vein of rats to induce nephrotic syndrome. After adriamycin treatment, the abatacept group rats were given abatacept (0.5 mg/kg) once by intraperitoneal injection on day 14. In addition, the prednisone group rats were given prednisone (12.5 mg/kg) daily consecutively by gavage from day 14 to day 21. Blood, urine, and kidney tissue specimens were collected when sacrificed on day 21. The 24-hour urinary protein, serum albumin, cholesterol, creatinine, and urea nitrogen were then detected. An enzyme-linked immunosorbent assay was used to determine the level of urine CD80 and serum IL-17. Flow cytometry was used to investigate the prevalence of circulating Treg. Hematoxylin-eosin staining and electron microscopy were used for a renal histological study. Immunofluorescence staining was performed to confirm the CD80 expression of renal tissue.

**Results:**

The 24-hour urinary protein of the abatacept group was significantly lower than that of the prednisone group and the AIN group. The level of urine CD80 of the abatacept group was significantly lower than that of the AIN group. Compared with the AIN group and the prednisone group, the circulating Treg prevalence of the abatacept group was significantly higher, while the level of serum IL-17 was lower. A negative kidney staining of CD80 expression was demonstrated in each group in this study. The 24-hour urinary protein had a negative correlation with the circulating Treg prevalence and Treg/IL-17 and a positive correlation with the urine CD80 and serum IL-17. Urinary CD80 had a positive correlation with serum IL-17 and no correlation with the circulating Treg prevalence.

**Conclusions:**

CTLA4-Ig abatacept can reduce proteinuria of adriamycin-induced nephropathy rats, possibly at least partially as a result of regulating circulating Treg/IL-17. CTLA4-Ig abatacept could be a promising regimen for idiopathic nephrotic syndrome.

## 1. Introduction

Idiopathic nephrotic syndrome (INS) is a highly prevalent glomerular disease that is characterized by massive proteinuria, hypoalbuminemia, hypercholesterolemia, and edema in childhood. Minimal change disease (MCD) and focal and segmental glomerulosclerosis (FSGS), considered podocytopathy, are the most common renal histopathologies. Although most patients experience a favorable response to glucocorticoids, nearly 80% of patients will relapse, and a long-term course of glucocorticoids or with a combination of immunosuppressants is often needed to maintain remission [1–3]. Moreover, some patients are resistant to current treatment regimens and then progress to end-stage renal disease (ESRD) [4]. Therefore, the exploration of new treatment options is of great clinical importance.

In recent years, the clinical application of cytotoxic T-lymphocyte-associated agent 4-immunoglobulin fusion protein (CTLA4-Ig) abatacept in proteinuric kidney disease cases has highlighted a new therapeutic option for INS [5–9]. Yu and colleagues first reported five patients with primary or recurrent FSGS, who showed a positive response to abatacept [5]. The same effective results were also subsequently determined in other MCD and FSGS patients [6–9]. However, another four FSGS patients who received abatacept still had persistent proteinuria [6]. A prospectively study of nine patients with recurrent FSGS after transplant demonstrated the same resistance to either abatacept or belatacept [10]. In addition, there was no change in proteinuria nor in creatinine in another case experience of abatacept [11]. It seems that the efficacy of CTLA4-Ig still remains to be clarified in nephrotic syndrome (NS).

CTLA4-Ig abatacept is an inhibitor that targets CD80 (also known as B7.1) and is currently used for rheumatoid arthritis (RA). CD80 is a T cell costimulator molecule expressed on antigen-presenting cells (APCs). The binding of CD80 to its receptor CD28 on T cells plays a key role in T cell activation, which can be inhibited by CTLA4 due to its competitive binding to CD80 [12]. The clinical application of abatacept in NS recently is largely due to a positive finding of CD80 expressed in podocytes in genetic, drug-induced, immune-mediated, and bacterial toxin-induced experimental kidney diseases, as well as in a few renal specimens from NS patients. This contributes to a reorganization of the podocyte actin cytoskeleton and damage to the podocyte structure leading to proteinuria. Abatacept prevents this upregulation, leading to podocyte protection and the remission of proteinuria [5, 6, 13–15]. However, the positive results have been challenged by a lack of reproducibility of CD80 staining in podocytes [16–18]. Therefore, the exact mechanism of the clinical response to CTLA4-Ig in NS remains a subject of debate.

Historically, INS has been considered to be related to T cell dysregulation [19]. An imbalance in the regulation of the regulate T cells (Treg)/helper T17 cells (Th17) has drawn particular attention as key players in the pathogenesis of INS for years. Obviously increased Th17, as well as its secreted factor, such as IL 17, and decreased Treg have been suggested not only in clinical studies but also in experimental models of active NS to contribute to podocyte injury, resulting in proteinuria. In addition, induced Treg has been shown to be able to induce regression of nephropathy [20–22]. Moreover, an ineffective circulating Treg response or dysfunction of autoregulatory mechanisms resulting in the inability of podocytes to express CTLA4 is hypothesized to cause increased CD80 expression and podocyte motility [23, 24]. Therefore, regulating or rebalancing Treg/Th17 is considered to be a potential therapeutic approach for INS. To the best of our knowledge, there are few studies that have focused on circulating T cell dysregulation, especially an imbalance in Treg/Th17 of NS subjected to CTLA4-Ig. Based on the strong evidence supporting the role of T cells in the pathogenesis of INS, it is hypothesized that blocking T cell activation with abatacept ameliorates proteinuria of NS by regulating circulating Treg/Th17.

## 2. Materials and Methods

### 2.1. Animal Studies

All of the animal experiments were performed according to institutional ethical committee guidelines and were approved by the Animal Ethics Committee of the Second Xiangya Hospital, Central South University. Experiments were conducted in accordance with the National Institutes of Health “Guide for the Care and Use of Laboratory Animals”. All efforts were made to minimize suffering.

Healthy male Sprague-Dawley (SD) rats of clean grade weighing between 180 and 200 g were used in this study. After adaptive feeding for one week, rats without proteinuria were randomly divided into the normal group (*n* = 8), the AIN group (*n* = 8), the abatacept group (*n* = 8), and the prednisone group (*n* = 8). An optimized dose of 6.5 mg/kg body weight of doxorubicin hydrochloride (adriamycin: ADR, Sigma, USA) or an equal volume of saline as normal was injected once via the tail vein in each rat. For the abatacept group, abatacept (0.5 mg/kg, R&D, USA) was given once by intraperitoneal injection on day 14 after ADR treatment. For the prednisone group, prednisone (12.5 mg/kg) was given daily consecutively by gavage from day 14 to day 21 after ADR treatment. All of the rats were sacrificed at the end of experiment on day 21 after ADR treatment. The blood, urine, and kidneys were harvested for further analysis.

### 2.2. Serum Albumin, Cholesterol, Creatinine, and Urea Nitrogen Analyses

Serum albumin, cholesterol, creatinine, and urea nitrogen were analyzed using an automated chemistry analyzer (FAITH-1000).

### 2.3. Urine Analysis

For urine samples, protein was measured using the Coomassie Brilliant Blue method, and creatinine concentrations were measured using an automated chemistry analyzer (FAITH-1000).

### 2.4. Urinary CD80 Measurements

Urinary CD80 was measured using a commercially available enzyme-linked immunosorbent assay (ELISA) kit (Huamei Life Science, Tianjin, China), which was adjusted for urinary creatinine (Ucr) excretion.

### 2.5. Treg Cell Analysis Using Flow Cytometry

Peripheral blood mononuclear cells (PBMCs) were obtained using the standard procedure. The Treg analysis was determined using a rats Treg staining kit (eBioscience, USA) according to the manufacturer's instructions. In brief, PBMCs were incubated with FITC conjugated anti-CD4 and PE-conjugated anti-CD25 antibodies at 4°C for 30 minutes. After CD4 and CD25 surface staining, the cells (1×10^6^/ml) were washed twice, fixed, and permeabilized at 4°C for 30 minutes in the dark. They were then stained intracellularly using the APC-conjugated anti-FOXP3 antibody (eBioscience, USA). Then, the cells were detected using a flow cytometer (Beckman, USA). The results are presented as a percentage of positive cells.

### 2.6. Serum IL-17 Analysis

Serum IL-17 was measured using a commercially available enzyme-linked immunosorbent assay (ELISA) kit (Huamei Life Science).

### 2.7. Kidney Morphological Analysis

Mouse kidneys were fixed in 4% paraformaldehyde and embedded with paraffin, and 3 *μ*m sections were stained with hematoxylin-eosin (HE). Electron microscopy studies were performed as previously described [22].

### 2.8. Immunofluorescence Staining of CD80 in the Renal Tissue Sections

A 3 *μ*m paraffin-embedded section attached to 0.01% poly-L-lysine-coated slices was prepared for immunofluorescence staining. To remove or decrease nonspecific binding, prepared slices were incubated using 10% goat serum for 60 minutes. Then, the sections were incubated with primary antibody monoclonal against CD80 (Abcam, USA) at 4°C overnight and washed three times in PBS (5 minutes each). This was followed by being incubated with a secondary antibody (Proteintech, USA) at 37°C for 90 minutes and washed three times in PBS (5 minutes each). Then, the DAPI staining solution was applied at 37°C for 10 minutes. After washing in PBS twice (5 minutes each), the sections were mounted with glycerin and observed under a fluorescence microscope (BA410E, Motic, Germany).

### 2.9. Statistical Analysis

The statistical analyses were performed using the SPSS 22.0 medical statistical software. Results were expressed as means ± standard deviations. The Kolmogorov-Smirnov test was used for the normal distribution test. A homogeneity of variance test was then performed. A variable transformation was performed for data without a normal distribution or homogeneity of variance. For data with a normal distribution and homogeneity of variance, a one-way analysis of variance (ANOVA) test was used for multiple group comparisons. For comparisons between two groups, an LSD-*t* test was performed. The two-variable relationship was analyzed using a Pearson correlation analysis. *P* < 0.05 was the level of statistical difference.

## 3. Results

### 3.1. Successful Modeling of AIN

**Figure 1:**
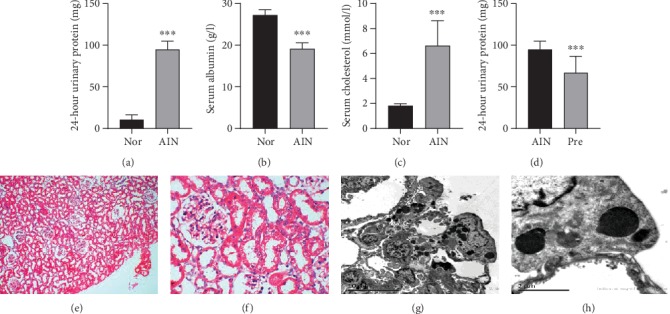
AIN was induced by tail vein injection of adriamycin in rats. It is characterized by proteinuria (a), hypoalbuminemia (b), and hypercholesterolemia (c). Proteinuria was reduced using prednisone (d). Data are shown as means ± SDs, *n* = 8. ^∗∗∗^*P* < 0.001*vs*. the Nor group. Nor: normal group; AIN: adriamycin-induced nephropathy group. HE staining showed glomerular morphology with minimal alterations ((d) original magnification ×100; (e) original magnification ×400); electron microscopy showed foot-process effacement ((g) original magnification ×2500; (f) original magnification ×12000).

According to the established protocol [25], SD rats were treated with 6.5 mg/kg body weight adriamycin to induce nephropathy. After adriamycin treatment, all of the rats manifested with proteinuria (3+) on day 14. On day 21 after adriamycin administration, 24-hour urinary protein in the AIN group obviously developed (94.93 ± 9.98 mg) compared with the normal group (10.61 ± 5.67 mg; *P* < 0.001; Figure 1(a)), while the serum albumin level was significantly lower (19.13 ± 1.46 g/l *vs.*27.25 ± 1.28 g/l, *P* < 0.001; Figure 1(b)), and the serum cholesterol level was significantly higher (6.64 ± 1.99 mmol/l *vs.*1.83 ± 0.16 mmol/l, *P* < 0.001; Figure 1(c)). In addition, proteinuria was reduced using prednisone (94.93 ± 9.98 mg *vs.*66.85 ± 19.79 mg, *P* < 0.001; Figure 1(d)). Minimal alternations of the glomerulus according to light microscopy and some focal foot-process effacement found by electron microscopy were observed (Figures 1(e)–1(h)). These data indicated a success in the establishment of the AIN modeling.

### 3.2. CTLA4-Ig Abatacept Reduced Proteinuria of the AIN Rats

**Figure 2:**
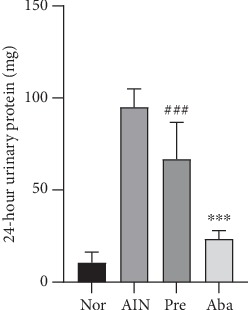
Abatacept reduced proteinuria of AIN rats. ^∗∗∗^*P* < 0.001*vs*. the AIN group and Pre group. ^###^*P* < 0.001*vs*. the AIN group. Nor: normal group; AIN: adriamycin-induced nephropathy group; Pre: prednisone group; Aba: abatacept group.

Multiple group comparisons of the 24-hour urinary protein in the four groups showed a statistical significance (*F* = 89.4111, *P* < 0.001). The 24-hour urinary protein in the abatacept group (23.49 ± 4.43 mg) was significantly lower than that in the AIN group (94.93 ± 9.98 mg) (*P* < 0.001) (Figure 2). Compared with the prednisone group (66.85 ± 19.79 mg), the 24-hour urinary protein in the abatacept group was significantly lower (*P* < 0.001; Figure 2). These data indicated that CTLA4-Ig abatacept administration reduced proteinuria in the AIN rats.

### 3.3. CTLA4-Ig Abatacept Decreased the Urinary CD80 of the AIN Rats

**Figure 3:**
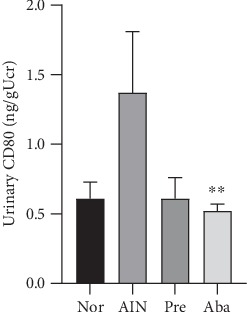
Abatacept decreased urinary CD80 of AIN rats. ^∗∗^*P* < 0.01*vs.* the AIN group. Nor: normal group; AIN: adriamycin-induced nephropathy group; Pre: prednisone group; Aba: abatacept group.

Multiple group comparisons of the urinary CD80 adjusted for urinary creatinine excretion in the four groups showed a statistical significance (*F* = 18.413, *P* < 0.001). The concentration of urinary CD80 in the AIN group (1.37 ± 0.44 ng/gUcr) was significantly higher than that in the normal group (0.61 ± 0.12 ng/gUcr) (*P* < 0.01). In addition, the concentration of urinary CD80 in the abatacept group (0.52 ± 0.05 ng/gUcr) and the prednisone group (0.61 ± 0.15 ng/gUcr) was significantly lower than that in the AIN group (*P* < 0.01). A comparison of urinary CD80 between the abatacept group and the prednisone group and between the abatacept group and the normal group showed no statistical significance (Figure 3).

### 3.4. CTLA4-Ig Abatacept Increased the Circulating Treg Prevalence and Decreased the Serum Concentration of IL-17 and Regulated Treg/IL-17 in the AIN Rats

**Figure 4:**
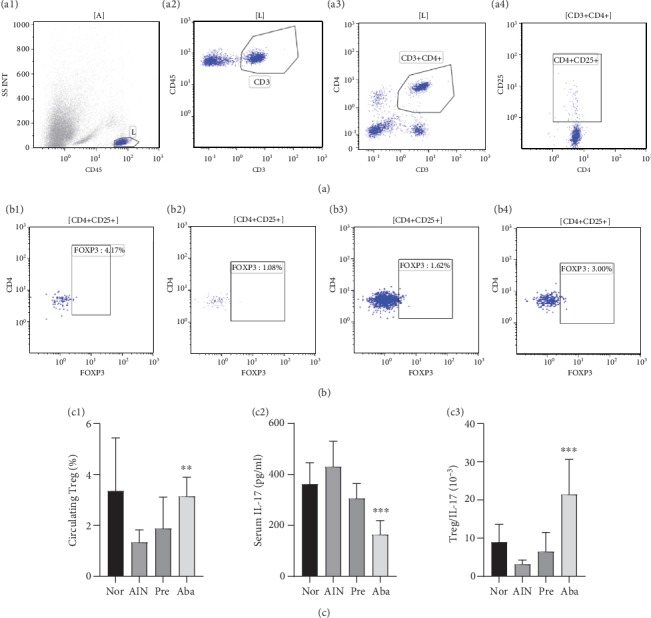
Abatacept regulated circulating Treg and IL-17 of AIN Rats. (a) CD3/CD45/SSC double-gating method was used for absolute count of CD3+CD45+FOXP3+ Treg cells. (b1) Treg of the normal group. (b2) Treg of the AIN group. (b3) Treg of the prednisone group. (b4) Treg of the abatacept group. (c1) Abatacept increased circulating Treg prevalence. ^∗∗^*P* < 0.01*vs.* the AIN group and *P* < 0.05*vs.* the Pre group. (c2) Abatacept decreased serum IL-17. ^∗∗∗^*P* < 0.001*vs.* the AIN group and Pre group. (c3) Abatacept regulated Treg/IL-17. ^∗∗∗^*P* < 0.001*vs.* the AIN group and Pre group. Nor: normal group; AIN: adriamycin-induced nephropathy group; Pre: prednisone group; Aba: abatacept group.

The CD3/CD45/SSC double-gating method was used for an absolute count of the CD3+CD45+FOXP3+Treg cells (Figure 4). Multiple group comparisons of the circulating Treg prevalence in the four groups showed a statistical significance (*F* = 5.36, *P* < 0.01; Figure 4(c1)). The circulating Treg prevalence in the abatacept group was significantly higher than that in the AIN group (3.14 ± 0.75% *vs.*1.33 ± 0.49%, *P* < 0.01) and that in the prednisone group (3.14 ± 0.75% *vs.*1.88 ± 1.23%, *P* < 0.05; Figure 4(c1)). However, there was no statistical significance in the circulating Treg prevalence between the abatacept group and the normal group (3.14 ± 0.75% *vs.*3.35 ± 2.09%, *P* > 0.05; Figure 4(c1)).

The concentration of serum IL-17 that is primarily secreted by Th17 cells in the peripheral blood represented the percentage of blood Th17 cells to a certain extent. Multiple group comparisons of the concentration of serum IL-17 in the four groups showed a statistical significance (*F* = 17.153, *P* < 0.01; Figure 4(c2)). The concentration of serum IL-17 in the abatacept group rats was significantly lower than that in the prednisone group (163.86 ± 55.28 pg/ml *vs.*305.64 ± 58.48 pg/ml, *P* < 0.001), in the AIN group (163.92 ± 59.71 pg/ml *vs.*429.86 ± 101.07 pg/ml, *P* < 0.001), and in the normal group (163.92 ± 59.71 pg/ml *vs.*361.83 ± 84.48 pg/ml, *P* < 0.001) (Figure 4(c2)).

Multiple group comparisons of Treg/IL-17 in the four groups showed a statistical significance (*F* = 13.637, *P* < 0.001). Treg/IL-17 in the abatacept group rats was significantly higher than that in the prednisone group ((21.40 ± 9.20)×10^−3^*vs.* (6.44 ± 5.08)×10^−3^, *P* < 0.001), in the AIN group ((21.40 ± 9.20) × 10^−3^*vs.*(3.16 ± 1.10) × 10^−3^, *P* < 0.001), and in the normal group ((21.40 ± 9.20) × 10^−3^*vs.*(8.95 ± 4.71) × 10^−3^, *P* < 0.001; Figure 4(c3)).

### 3.5. CTLA4-Ig Abatacept Preserved Renal Function of the AIN Rats and Maintained AIN Rat Kidney Morphology

**Figure 5:**
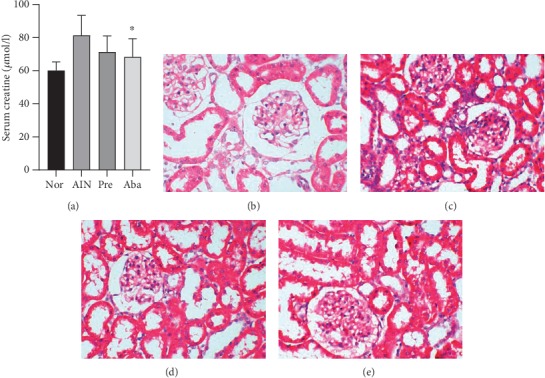
(a) Abatacept preserved renal function of AIN rats. ^∗^*P* < 0.05*vs.* the AIN group. Nor: normal group; AIN: adriamycin-induced nephropathy group; Pre: prednisone group; Aba: abatacept group. (b–e): abatacept maintained AIN rat kidney morphology. (b). Renal HE staining of the normal group. (c). Renal HE staining of the AIN group. (d). Renal HE staining of the prednisone group. (e). Renal HE staining of the abatacept group (original magnification ×400).

As shown in Figure 5(a), the level of serum creatinine (Scr) in the abatacept group was significantly lower than that in the AIN group (68.25 ± 11.05 *μ*mol/l *vs.*81.38 ± 11.98 *μ*mol/l, *P* < 0.05), while no statistical difference was found between the abatacept group and the normal group (68.25 ± 11.05 *μ*mol/l *vs.*60.13 ± 5.17 *μ*mol/l, *P* > 0.05). This indicated that CTLA4-Ig abatacept administration preserved the renal function of the AIN rats.

The renal specimen histopathology of the AIN rats showed mild focal mesangial prominence and interstitial inflammatory cell infiltration under light microscopy (Figure 5(c)), while the CTLA4-Ig abatacept administered rats' kidneys showed less mesangial proliferation (Figure 5(e)).

### 3.6. CD80 Was Not Found in the Kidneys of the AIN Rats

**Figure 6:**
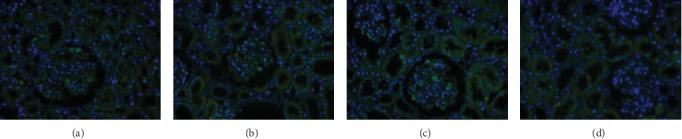
Negative CD80 staining of the kidneys in each group of rats. (a). CD80 immunofluorescence staining of the kidneys in the normal group. (b). CD80 immunofluorescence staining of the kidneys in the AIN group. (c). CD80 immunofluorescence staining of the kidneys in the prednisone group. (d). CD80 immunofluorescence staining of the kidneys in the abatacept group (original magnification ×400).

No positive CD80 staining was found in the renal tissue of each group of rats, as shown in Figure 6. This result means that CD80 may have likely not been expressed in the kidneys of the AIN rats.

### 3.7. 24-Hour Urinary Protein Was Correlated with Circulating Treg Prevalence, Serum IL-17, Treg/IL-17, and Urinary CD80

**Figure 7:**
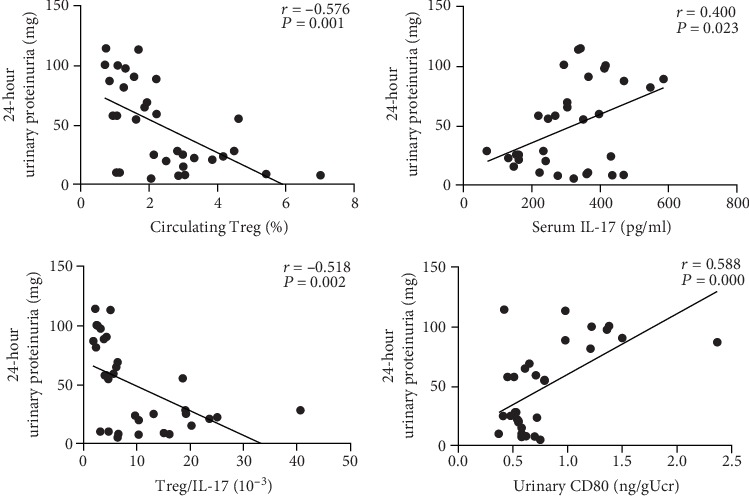
24-hour urinary protein showed a negative correlation with circulating Treg prevalence and Treg/IL-17 and a positive correlation with serum IL-17 and urinary CD80.

The Pearson correlation analysis showed that the 24-hour urinary protein had a negative correlation with the circulating Treg prevalence (*r* = −0.576, *P* = 0.001) and Treg/IL-17 (*r* = −0.581, *P* = 0.002) and a positive correlation with urinary CD80 (*r* = 0.588, *P* = 0.000) and serum IL-17 concentration (*r* = 0.400, *P* = 0.023; Figure 7).

### 3.8. Urinary CD80 Has a Correlation with Serum IL-17 and Treg/IL-17 and No Correlation with Circulating Treg Prevalence

**Figure 8:**
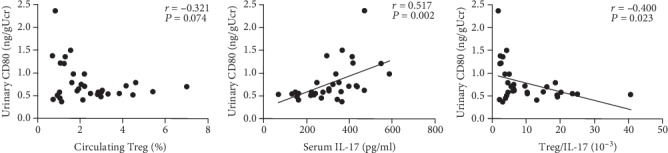
Urinary CD80 showed a positive correlation with serum IL-17 and Treg/IL-17, whereas there was no correlation with the circulating Treg prevalence.

The Pearson correlation analysis showed that urinary CD80 had a positive correlation with the serum IL-17 concentration (*r* = 0.517, *P* = 0.002) and Treg/IL-17 (*r* = 0.400, *P* = 0.023) and no correlation with the circulating Treg prevalence (*r* = −0.321, *P* = 0.074; Figure 8).

## 4. Discussion

In this study, the efficacy of CTLA4-Ig abatacept and its possible mechanism in the reduction of proteinuria in a murine model of AIN were evaluated. The three principle findings are as follows: (1) CTLA4-Ig abatacept ameliorated proteinuria in AIN rats, (2) CTLA4-Ig abatacept decreased urine CD80, and (3) CTLA4-Ig abatacept regulated circulating Treg/IL-17.

INS is manifested with massive proteinuria, hypoalbuminemia, hypercholesterolemia, and generalized edema, and its common renal histopathology is MCD or FSGS. AIN is an established animal model that effectively mimics this pathophysiology [25]. In this study, it was also found that the AIN rats had similar proteinuria, hypoalbuminemia, hypercholesterolemia, minimal glomerular pathomorphological changes, and foot processes effacement that were alleviated using prednisone (Figure 1). More importantly, an imbalance in Treg/Th17 and the elevation of urinary CD80 were also confirmed in this study, which is consistent with the results of previous studies [21, 26]. Therefore, this model is very suitable for the study of the efficacy of CTLA4-Ig and its possible mechanism in INS.

In clinical practice, MCD is often considered to be sensitive to glucocorticoid therapy, but approximately 80% of cases experience relapses, and some remain steroid dependent or become steroid resistant, whereas FSGS is typically insensitive. Long-time steroids combined with immunosuppressants are conventional regimens, despite the side effects. In addition, frequent relapse and treatment-resistant INS are at great risk for progression to ESRD [4]. The clinical usage of CTLA4-Ig, such as abatacept and belatacept, in NS highlights a new therapeutic approach for INS [5–9]. Yu and colleagues first reported five patients with primary or recurrent FSGS who showed response to abatacept [5]. Thereafter, several steroid-resistant or steroid-dependent MCD and FSGS cases were reported to be sensitive to abatacept [6–9]. However, Garin and colleagues reported four FSGS cases who received abatacept and remained unchanged [6]. In addition, a prospective study of nine patients with recurrent FSGS after transplant conducted by Delville et al. demonstrated resistance to either abatacept or belatacept [10]. The effects of CTLA4-Ig in normalizing proteinuria in FSGS are controversial, but it appears to have beneficial effects on MCD. To the best of our knowledge, few studies have shown the effect of CTLA4-Ig for reducing proteinuria in experimental NS. The data obtained in this study demonstrated that a single-dose infusion of abatacept at the onset of NS is effective to reduce proteinuria abruptly. In addition, it was shown to be much more effective compared with prednisone therapy in the early stage of the disease (Figure 2). Moreover, abatacept significantly attenuated AIN rat renal damage, as abatacept administration preserves renal function of AIN rats and maintains their kidney morphology (Figure 5). Therefore, our intervention arm data indicated that CTLA4-Ig abatacept was effective in proteinuria normalization of AIN rats, suggesting that abatacept would be a promising regimen for INS, at least in MCD.

As is well known, the target of CTLA4-Ig abatacept is CD80. CD80 is considered a potential therapeutic target for INS due to experiments in vitro and in vivo showing that the upregulation of CD80 on podocyte can lead to proteinuria [13, 14]. In addition, urinary CD80 excretion increases in relapse INS, especially in MCD, whereas serum CD80 remains unchanged [15, 27, 28]. In addition, positive staining of CD80 in renal specimens of both relapse MCD and FSGS further indicates a possible pathogenesis of podocyte CD80 in INS, and it is utilized as a biomarker to identify patients who may benefit from abatacept [5, 6, 15]. To explore the mechanisms of the beneficial effects of abatacept in the AIN model, urinary CD80 and renal CD80 expressions were further investigated. Consistent with previous studies [26, 27], the data from this study also demonstrated an elevation of urinary CD80 and a positive correlation with proteinuria (Figures 3 and 7). In addition, the proteinuria reduction in AIN rats was accompanied by decreased urinary CD80 after the CD80 blockage abatacept infusion (Figure 3). However, interestingly, in this study, negative staining of CD80 by immunofluorescence in the renal specimens of both normal rats and AIN rats was found (Figure 6). This negative result was exactly the same as that demonstrated by Baye and colleagues who showed that CD80 was not expressed on podocyte cells exposed to lipopolysaccharides *in vitro* and *in vivo* [29]. This result also occurred in other mouse models of podocyte injury, including treatment with adriamycin, a lupus prone model (NZB/W F1), and subtotal nephrectomy [29]. Moreover, the positive staining of CD80 on podocyte cells has not been reproduced in other studies, including a large number of MCD and FSGS studies, even though more than one commercially available anti-CD80-antibody was used on both frozen and paraffin-embedded renal biopsy samples [16–18]. Therefore, the negative findings in this study of podocyte CD80 indicate that it is still controversial that podocyte CD80 is the real target of abatacept for INS. Further study is required to confirm the existence of podocyte CD80 and its exact action in INS.

It is a known fact that CTLA-4 Ig abatacept blocks CD28-mediated T cell activation by binding to the costimulatory B7 ligands CD80/CD86 on APCs, and it is currently used for RA and other autoimmune diseases. Studies have already confirmed that abatacept modulated Treg/Th17 in RA patients [30, 31]. As dysregulation of Treg/Th17 is considered to be a key player in the pathogenesis of INS, peripheral Treg cells (Tregs) and serum IL-17 were further detected in this study, which represents peripheral Th17 cells to a certain extent. Consistent with the previous research [20, 21], increased serum IL-17 and decreased peripheral Tregs in the AIN rats were also demonstrated in this study (Figure 5), suggesting an excessive T cell activation and a decreased anti-inflammatory milieu in NS. When treating AIN rats with abatacept, serum IL-17 decreased and peripheral Tregs elevated (Figure 5). In addition, a correlation analysis revealed that the 24-hour urinary protein had a negative correlation with the circulating Treg prevalence and Treg/IL-17 and a positive correlation with serum IL-17 (Figure 7). Hence, these results indicate that abatacept ameliorated proteinuria in AIN rats probably by regulating Treg/IL-17. Hence, the more noticeable amelioration of Treg/IL-17 in abatacept therapy may contribute to less proteinuria compared with prednisone therapy.

It is certain that abatacept is able to reduce serum IL-17 through its physical blockage of CD28 signaling in the priming of pathogenic T effort cells (especially Th17 cells) [30, 32]. In addition, abatacept treatment may therefore reduce the amount of CD28-mediated signaling to Tregs and cause a decrease in their population [32, 33]. Conversely, increased peripheral Tregs were found after abatacept infusion. This confusion of a significant increase in Tregs has also been revealed before in papers that have investigated RA patients subjected to abatacept [31, 34]. In addition, CD28^−/−^ Treg-specific mice had only a 25%-30% reduction in their thymic Tregs and no significant reduction in the periphery, indicating that CD28 signaling is not a unique modulator of peripheral Treg homeostasis [35]. In addition, according to the massive data regarding abatacept treatment, it may increase the suppressor function and have some effect on the population size of Tregs, but no strong conclusions can be made [32]. The exact mechanism of the efficacy of abatacept and its effect on Tregs are not clear and can be conflicting. Perhaps, subpopulations of Tregs should be assessed to obtain better associations regarding abatacept-induced effects [32].

Urinary CD80 has been shown to have a positive correlation with serum IL-17, but no correlation with Treg (Figure 8). In addition, if the negative expression of CD80 in AIN rat kidneys is correct, urinary CD80 is likely secreted from circulation, also indicating an excessive T cell activation in AIN rats. However, there is a limitation in this study because this study was not designed to detect serum CD80, as no change has been reported before in INS [15]. Hence, this aspect requires further study.

In summary, this study demonstrated a sufficiency in normalizing proteinuria by CTLA4-Ig abatacept as a result of a modulation of Treg/IL-17 in experimental NS. Clinical randomized trials using CTLA4-Ig in INS are still required to validate its beneficial effects [36]. The exact mechanism of the efficacy of abatacept in INS, especially the effect on Tregs and its biomarker to identify patients who may benefit from abatacept, requires further investigation.

## 5. Conclusion

The results of this study showed that there is potential for CTLA4-Ig abatacept to reduce proteinuria in AIN rats, and the possible explanation for this effect may be the result of the regulation of peripheral Treg/IL-17. CTLA4-Ig abatacept could therefore be a promising regimen for INS.

## Data Availability

The data used to support the findings of this study are included within the article.
